# A Hyperstabilizing Mutation in the Base of the Ebola Virus Glycoprotein Acts at Multiple Steps To Abrogate Viral Entry

**DOI:** 10.1128/mBio.01408-19

**Published:** 2019-07-09

**Authors:** J. Maximilian Fels, Jennifer S. Spence, Robert H. Bortz, Zachary A. Bornholdt, Kartik Chandran

**Affiliations:** a Department of Microbiology and Immunology, Albert Einstein College of Medicine, Bronx, New York, USA; b Mapp Biopharmaceutical Inc., San Diego, California, USA; University of Pittsburgh School of Medicine

**Keywords:** Ebola, cell entry, ebolavirus, filovirus, fusion activation, glycoprotein, thermostability, viral entry, viral membrane fusion

## Abstract

Ebola virus (EBOV) causes highly lethal disease outbreaks against which no FDA-approved countermeasures are available. Although many host factors exploited by EBOV for cell entry have been identified, including host cell surface phosphatidylserine receptors, endosomal cysteine proteases, and the lysosomal cholesterol trafficking protein NPC1, key questions remain. Specifically, late entry steps culminating in viral membrane fusion remain enigmatic. Here, we investigated a set of glycoprotein (GP) mutants previously hypothesized to be entry defective and identified one mutation, R64A, that abolished infection with no apparent impact on GP expression, folding, or viral incorporation. R64A profoundly thermostabilized EBOV GP and rendered it highly resistant to proteolysis *in vitro*. Forward-genetics and cell entry studies strongly suggested that R64A’s effects on GP thermostability and proteolysis arrest viral entry at least at two distinct steps: the first upstream of NPC1 binding and the second at a late entry step downstream of fusion activation. Concordantly, toremifene, a small-molecule entry inhibitor previously shown to bind and destabilize GP, may selectively enhance the infectivity of viral particles bearing GP(R64A) at subinhibitory concentrations. R64A provides a valuable tool to further define the interplay between GP stability, proteolysis, and viral membrane fusion; to explore the rational design of stability-modulating antivirals; and to spur the development of next-generation Ebola virus vaccines with improved stability.

## Abstract (precis)

Ebola virus is a medically relevant virus responsible for outbreaks of severe disease in western and central Africa, with mortality rates reaching as high as 90%. Despite considerable effort, there are currently no FDA-approved therapeutics or targeted interventions available, highlighting the need of development in this area. Host-cell invasion represents an attractive target for antivirals, and several drug candidates have been identified; however, our limited understanding of the complex viral entry process challenges the development of such entry-targeting drugs. Here, we report on a glycoprotein mutation that abrogates viral entry and provides insights into the final steps of this process. In addition, the hyperstabilized phenotype of this mutant makes it useful as a tool in the discovery and design of stability-modulating antivirals and next-generation vaccines against Ebola virus.

## INTRODUCTION

Ebola virus (EBOV) belongs to the family *Filoviridae* of enveloped viruses with negative-strand RNA genomes and is responsible for sporadic outbreaks of a highly lethal hemorrhagic fever. Ebola virus disease (EVD) is characterized by symptoms such as vomiting, diarrhea, fever, and various forms of coagulopathy, and there are currently no FDA-approved vaccines or therapeutics available (1).

Considerable progress has been made in understanding the molecular mechanisms of host cell invasion by EBOV and other filoviruses, including the identification of their shared endosomal receptor Niemann-Pick C1 (NPC1), which is strictly required for infection (2–4). Other host factors required for filovirus entry include members of the cysteine cathepsin family of endosomal proteases, cathepsin B and cathepsin L (CatB and CatL, respectively), which play virus- and cell type-dependent roles (5–7). These enzymes cleave the viral glycoprotein (GP) at the β13–14 loop (residues 189 to 214) in order to allow NPC1 binding and subsequent steps in viral membrane fusion. GP, a class I viral fusion protein consisting of the subunits GP1 and GP2, is displayed on viral surfaces as a trimer of heterodimers. GP1 mediates viral attachment to the cell surface and intracellular receptor and regulates the prefusion conformation of GP2. GP2 drives the membrane merger events that result in viral membrane fusion in a manner analogous to that of HA2 of influenza A virus or gp41 of HIV-1 (8, 9). However, the exact cue that triggers the large-scale reorganization of GP2 required for membrane fusion remains unknown. Because proteolytically primed GP is still sensitive to inhibition of infection by the broad-spectrum cysteine protease inhibitor E-64, it has been suggested that an additional proteolytic event is required to generate fully fusogenic GP (10, 11). Another proposed fusion trigger is reduction of the disulfide bond between the two GP subunits (GP1 and GP2). This is supported by the observation that mild reduction increases the ability of GP to associate with membranes *in vitro* (12).

A previous study of GP function by Manicassamy et al. used alanine scanning of GP1 to reveal a set of residues that reduced infection by >90% when mutated, without impacting GP expression, folding, or incorporation into HIV pseudotypes (13). Based on the clustered location of these residues at the base of GP1, we hypothesized that they have a functional role in entry, potentially related to viral fusion triggering.

Here, we reconstituted five GP mutants (with R64A, L63A, L57K, L57A, and D55A mutations) as vesicular stomatitis virus (VSV) pseudotypes to investigate their impact on viral entry. One of these mutations, R64A, resulted in a severe decrease in infectivity that could not be explained by an inherent defect in endosomal trafficking, NPC1 binding, or fusion loop deployment. Instead, our findings provide evidence that R64A greatly increases the thermal stability of GP, leading to a decrease in proteolytic sensitivity and blocks at multiple steps in the entry pathway. We further identified a panel of second-site reversion mutations in the GP base that could rescue the infectivity of VSV-GP(R64A) and partially restore the thermal sensitivity of GP. Our findings provide mechanistic insights into the process by which filovirus GP mediates viral membrane fusion and cytoplasmic escape.

## RESULTS

### Mutation R64A in the EBOV GP1 base abrogates viral entry.

**FIG 1:**
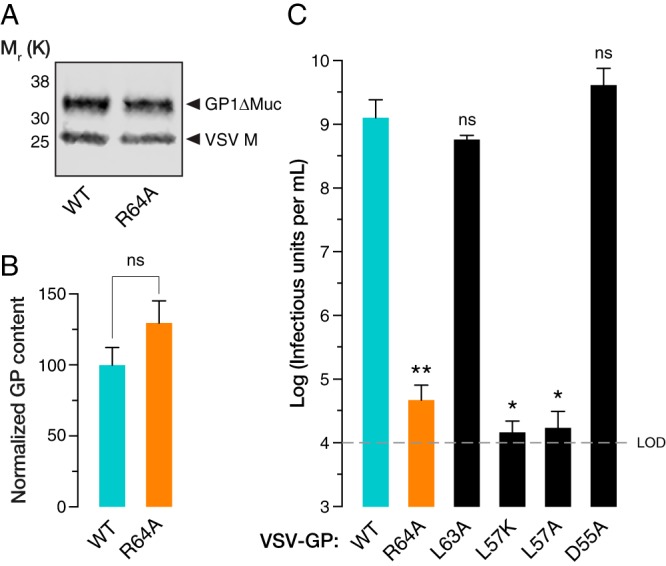
(A) Quantitative Western blotting of VSV pseudotypes bearing GP(WT) or GP(R64A) showing similar levels of EBOV GP1 and VSV M proteins. M_r_ (K), relative molecular weight (in thousands). (B) Quantification of relative GP/M signals for WT and R64A VSV pseudotypes, showing no significant difference in GP incorporation. (C) Mean viral titers of WT and mutant GP pseudotypes from two independent experiments (*n* = 6). GP mutants were tested for statistically significant loss of infectivity compared to that of the WT using unpaired one-tailed *t* tests (*, *P* < 0.033; **, *P* < 0.002). The R64A mutation leads to a >4-logarithmic-unit reduction in viral titer (limit of detection [LOD] = 1 × 10^–4^ IU/ml), while mutations L63A and D55A have no significant impact on infectivity. Mutations at position 57 also reduce infectivity but are associated with poor GP incorporation (data not shown) and were therefore not investigated further.

A comprehensive alanine scan of the EBOV GP1 subunit performed by Manicassamy and coworkers (13) identified residues critical for EBOV GP-dependent entry that did not impact GP expression or incorporation into pseudotyped HIV particles. Although some of the critical residues mapped to the NPC1 receptor-binding site (RBS) of GP1, a distinct set (R64A, L63A, L57K, L57A, D55A) were found in a region of the GP1 base subdomain with no known function (14). To elucidate the effects of these mutations on viral entry, we reconstituted them into VSV pseudotypes expressing enhanced green fluorescent protein (eGFP) as an infection marker, as described previously (15). GP(R64A) was incorporated into VSV particles at high levels (Fig. 1A and B) yet caused a dramatic reduction in viral infectivity (Fig. 1C). Therefore, we selected this mutant for further analysis. Mutations at position 57 likewise abolished infectivity but were found to reduce GP incorporation into VSV particles (data not shown), and they were therefore not studied further.

### GP(R64A) is highly resistant to proteolysis by thermolysin.

**FIG 2:**
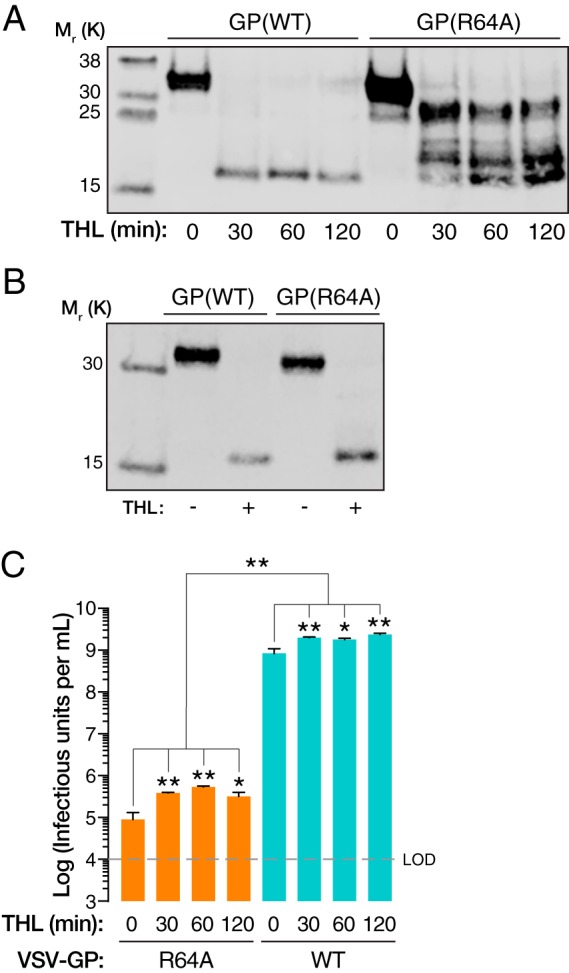
(A) After incubation with THL and deglycosylation by PNGase F, GP cleavage products of GP(WT) and GP(R64A) were analyzed by Western blotting. GP(WT) is readily and completely converted to a species with a relative molecular weight of ∼17K, while GP(R64A) shows marked resistance to proteolysis. (B) Upon prolonged incubation with THL at higher concentrations, GP(R64A) can be converted to a single cleavage product. (C) Mean viral titers of VSV-GP(WT) and VSV-GP(R64A) following THL treatment from one representative experiment (*n* = 3), showing that *in vitro* cleavage of GP(R64A) cannot fully rescue infection. For both VSV-GP(WT) and -GP(R64A), there is a small but statistically significant increase in infectivity upon THL treatment. Under no condition is the infectivity of VSV-GP(R64A) restored to GP(WT) levels (*, *P* < 0.033; **, *P* < 0.002, by one-way ANOVA with Dunnett’s correction for multiple comparisons).

The proteolytic priming of GP by host cysteine proteases CatB and/or CatL is a prerequisite for EBOV entry (5, 6). Considering the location of R64 at the base of GP1, in proximity to the β13–14 loop, where this priming cleavage occurs, we next examined the capacity of GP(R64A) to undergo entry-related proteolytic cleavage. VSVs pseudotyped with wild-type GP [GP(WT)] or GP(R64A) were incubated with the bacterial protease thermolysin (THL), which can substitute for CatB in generating a proteolytically primed GP intermediate *in vitro* (6, 16). Following deglycosylation by treatment with peptide-*N*-glycosidase F (PNGase F), cleavage products were analyzed by Western blotting. GP1(WT) was completely converted to a form of GP1 that has a relative molecular weight of approximately 17,000 (GP1-17K; also referred to as GP1-19K prior to deglycosylation) within 30 min under these conditions, as reported previously (5, 6). In contrast, GP(R64A) was highly resistant to proteolysis, as evidenced by the persistence of several cleavage intermediates (Fig. 2A). Prolonged incubation of VSV-GP(R64A) with THL did afford extensive cleavage; however, the apparent molecular weight of the terminally cleaved GP(R64A) product is larger than that observed for GP(WT) (Fig. 2B). We hypothesize that this is an artifact of the charge difference induced by the R-to-A mutation, as amino acid sequence analysis through mass spectrometry of this product revealed no differences (data not shown). A partial, but statistically significant, rescue of viral infectivity was observed upon GP(R64A) cleavage, indicating that this mutation can block viral entry both at steps upstream and at steps downstream of the GP priming events recapitulated by *in vitro* protease treatment (Fig. 2C).

### Proteolytically cleaved GP(R64A) is able to bind NPC1.

**FIG 3:**
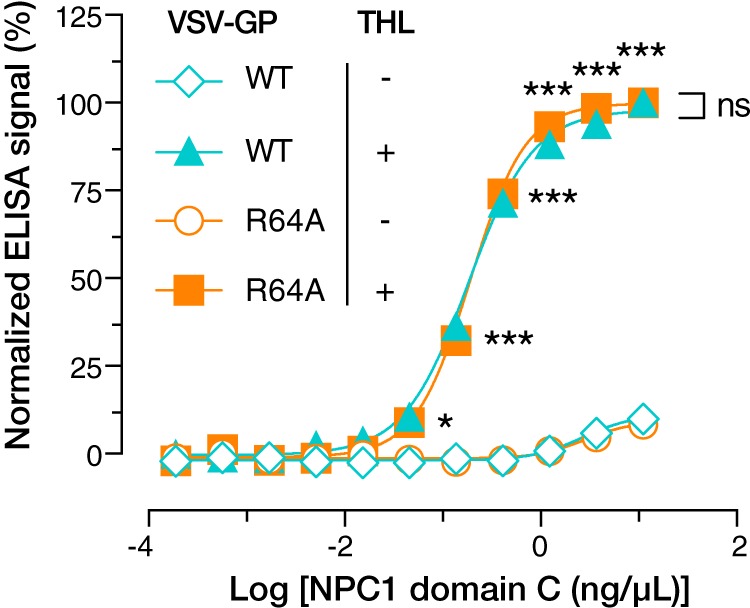
Normalized amounts of THL-cleaved VSV-GP(WT) and -GP(R64A) (as in Fig. 2B) were captured onto ELISA plates using EBOV MAb KZ52. Addition of soluble NPC1 domain C shows cleavage-dependent and equal levels of absorption to both GP(WT) and GP(R64A) (*, *P* < 0.033; **, *P* < 0.002; ***, *P* < 0.001, determined by unpaired *t* tests with Benjamini, Krieger, and Yekutieli correction for multiple testing). Means of results from six trials from two independent experiments with three technical replicates are shown.

We next assessed the capacity of uncleaved and *in vitro*-cleaved GP(R64A) to bind to soluble domain C of NPC1 by enzyme-linked immunosorbent assay (ELISA), as described previously (17). Although uncleaved GP(WT) and GP(R64A) did not bind to domain C, as expected, both could readily bind once cleaved (Fig. 3). These findings strongly suggest that the reduced infectivity of GP(R64A) does not stem from an intrinsic defect in its capacity, if cleaved, to engage the viral receptor in host cell endosomes.

### VSV-GP(R64A) displays an aberrant pattern of fusion activation in live cells.

**FIG 4:**
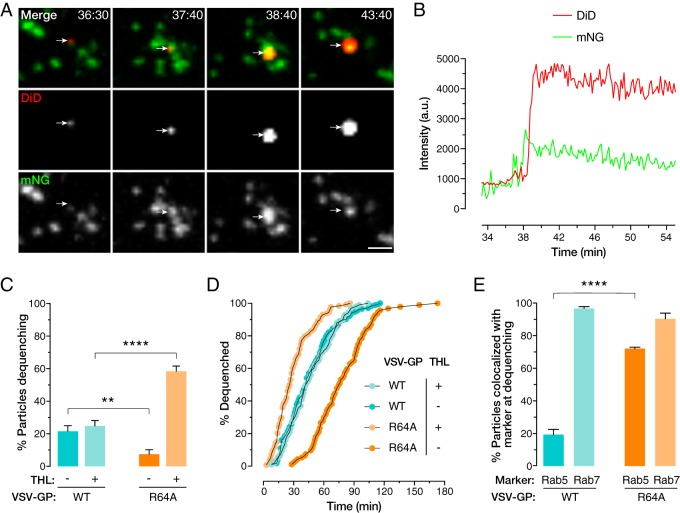
(A and B) Example of DiD-labeled VSV-GP(WT) lipid mixing event in a compartment positive for monomeric NeonGreen (mNG)-tagged NPC1 (A) and fluorescence intensity trace for the indicated tracked particle (B). a.u., arbitrary units. Bar, 5 μm. (C) Total percentage of cell-associated particles bearing GP(WT) or GP(R64A) undergoing lipid mixing. (D) Kinetics of GP(WT)- or GP(R64A)-mediated lipid mixing with and without GP precleavage. (E) Percentage of precleaved GP(WT) and GP(R64A) particles colocalized with the endosomal markers Rab5 and Rab7 at the time of lipid mixing.

In order to examine how the R64A mutation alters discrete events in EBOV entry, we performed live-cell microscopy and single-particle tracking. We previously showed that membrane labeling with self-quenching concentrations of the lipophilic dye DiD (1,1′-dioctadecyl-3,3,3′,3′- tetramethylindodicarbocyanine, 4-chlorobenzenesulfonate salt) could be used to uncouple proteolytic requirements for fusogenic GP activation from infection, in addition to enabling viral trafficking to be followed (11). Unlike VSV-GP(WT) (Fig. 4A and B), virions bearing EBOV GP(R64A) (11) were largely unable to engage in lipid mixing, as indicated by DiD dequenching, suggesting that this mutant is relatively fusion null within cells (Fig. 4C). This low level of lipid mixing is consistent with the diminished infection observed for VSV-GP(R64A) in infection assays. The GP(R64A) particles that underwent fusion triggering did so at a much lower rate than that of GP(WT) (Fig. 4D). Interestingly, complete cleavage of VSV-GP(R64A) with THL prior to infection enhanced lipid mixing activity, even compared to that of GP(WT). We note that a high concentration of THL and lengthy incubation period were necessary to produce this fusogenic form, which endosomal conditions are highly unlikely to replicate. While precleavage rescues fusogenicity, the same proteolytic treatment fails to restore infectivity (Fig. 2C). We have shown that EBOV GP can trigger membrane fusion within intermediate endosomes for a relatively small fraction of dequenching particles (11). Consistently with their faster lipid-mixing kinetics (Fig. 4D), approximately 70% of the VSV-GP(R64A) particles that dequenched did so in Rab5-positive compartments, compared to approximately 20% of the WT control, whereas colocalizations with Rab7 at the time of dequenching were similarly high for both viruses (Fig. 4E). These findings suggest that, in contrast to the precleaved version of VSV-GP(WT), the precleaved GP(R64A) mutant is predominantly triggered to fuse in intermediate endosomes and exhibits a lower threshold for fusion triggering. They also strongly suggest that the failure of most uncleaved VSV-GP(R64A) particles to undergo lipid mixing reflects an upstream block, likely at one or more endosomal cleavage steps that generate a primed intermediate capable of fusion activation. The lack of infectivity of viral particles bearing the precleaved GP(R64A) mutant indicates an additional block at a late step in entry, downstream of fusion activation (Fig. 2C).

### A forward-genetics strategy reveals compensatory mutations that rescue viral entry by GP(R64A).

**FIG 5:**
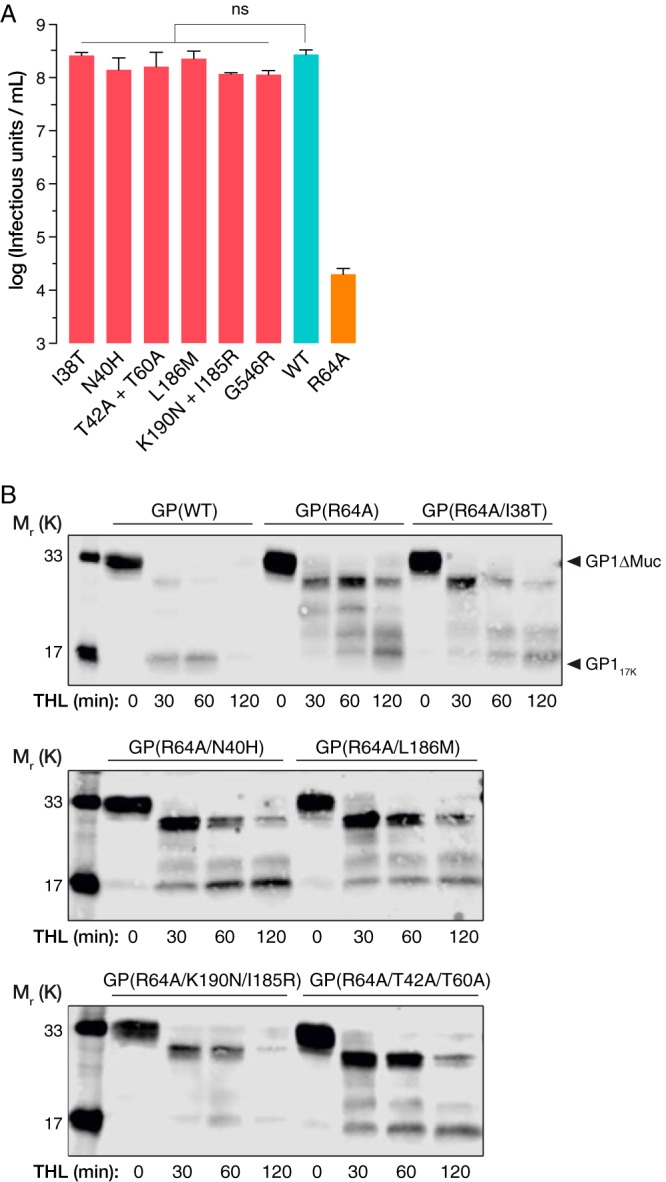
(A) Mean viral titers of recombinant VSV-GP(WT) and a set of R64A second-site revertants from two independent experiments (*n* = 5), showing full rescue of viral infectivity (there was no statistically significant difference in titer [ns], determined by one-way ANOVA with Dunnett’s correction for multiple testing). The titer of pseudotyped VSV-GP(R64A) is included for reference. (B) THL cleavage patterns, performed as in Fig. 2A, for a set of R64A second-site revertants, showing a variable but overall increased resistance to proteolysis in a comparison with GP(WT).

As a complement to the biochemical approaches described above, we employed a forward-genetics strategy to identify potential compensatory mutations in GP that can restore the infectivity of VSV-GP(R64A). Specifically, we attempted to rescue a recombinant VSV encoding GP(R64A) from cDNA (10, 18). To disfavor reversion to WT, the A64 codon (GCC) was engineered to require two point mutations to generate R64 revertants. Recombinant virus was successfully recovered from plasmid-transfected cell cultures and further amplified by passage of cell supernatants in Vero cells (see Materials and Methods for details). All viral clones isolated by plaque purification retained the R64A mutation, but each also bore a second- and/or third-site mutation (Table 1). Plaque-purified second-site revertants all grew to WT titers (Fig. 5A).

**TABLE 1:** List of R64A second-site revertants^*a*^

Second-site mutation(s)	GP subunit	CatB independent
I38T	GP1	Yes
N40H	GP1	Yes
T42A T60A	GP1	Yes
L186M	GP1	No
I185R K190N	GP1	Yes
G546R	GP2	No

a All revertants retained the R64A mutation but bore one or more additional mutations.

Because resistance to proteolytic processing *in vitro* is one of the defining features of GP(R64A), we evaluated the second-site revertants for this phenotype. Accordingly, we incubated recombinant VSV (rVSV)-GP(R64A) mutants with a set amount of THL for increasing amounts of time and analyzed GP cleavage products by Western blotting. Interestingly, the second-site mutations, on the whole, did not overtly reverse the resistance of GP(R64A) to THL cleavage, suggesting that the cleavage resistance phenotype observed *in vitro* does not correlate with GP(R64A)’s entry defect or its rescue by the second-site mutations (Fig. 5B).

### A subset of second-site mutations alter the protease requirement for infection.

**FIG 6:**
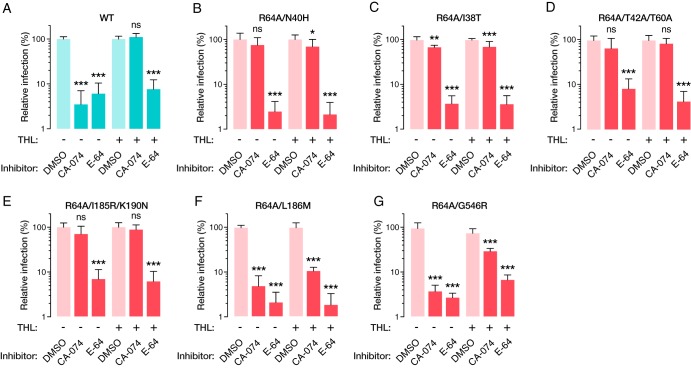
(A to F) Mean relative infection levels of THL-treated and native rVSV-GP(WT) and R64A second-site revertants, in the presence of CA-074, E-64, or a DMSO control. rVSV-GP(WT) is sensitive to E-64 under all conditions but escapes CA-074 block following THL treatment. R64A second-site revertants are likewise inhibited by E-64 but, with the exception of L186M and G546R, are largely CA-074 insensitive. Results from eight trials from four independent experiments with two technical replicates are shown. Infection levels following CA-074 or E-64 treatments were analyzed for statistical significance using one-way ANOVA with Dunnett’s correction for multiple comparisons (*, *P* < 0.033; **, *P* < 0.002; ***, *P* < 0.001).

Mutations at two of the amino acid residues identified in our second-site revertant selection (N40 and T42) have previously been shown to confer CatB independence upon GP-mediated infection (10). These residues form part of an NXT sequon, and their mutation was previously shown to abrogate N-linked glycosylation at N40. We hypothesized that the CatB independence conferred by the N40 and T42 mutations would also occur in the context of R64A, and that the remaining second-site mutations also rescued infectivity by changing the protease requirements for GP-mediated entry. Accordingly, we pretreated Vero cells with the cysteine protease inhibitor CA-074 under CatB-selective conditions established previously (5, 10) and then exposed them to uncleaved or THL-treated virus. As expected, infection by virus with uncleaved GP(WT) was potently inhibited by CA-074, whereas THL-treated virus was insensitive to the drug. Both the uncleaved and cleaved viruses remained sensitive to the broad-spectrum cysteine protease inhibitor E-64, as shown previously (5, 6, 10), consistent with their continued dependence on endosomal cysteine cathepsin activity for viral membrane fusion (Fig. 6A). In contrast to their WT counterpart, the second-site I38T, N40H, T42A T60A, and K190N I185R revertants were largely insensitive to CA-074 in both their cleaved and uncleaved states, indicating that they enter cells in a CatB-independent manner (Fig. 6B to E). Unexpectedly, the L186M mutation caused an increased dependence on CatB, as even the THL-treated virus was sensitive to CatB inhibition (Fig. 6F). G546R similarly shows a partial increased dependence on CatB (Fig. 6G). Thus, despite the lack of evidence for gross changes in THL-mediated proteolysis of GP *in vitro* (Fig. 5B), our findings do point to more-subtle changes in protease dependence induced by the second-site mutations. However, the divergent (CatB-hyperdependent) behavior of L186M, and to a lesser extent of G546R, suggests that the acquisition of CatB independence alone cannot fully explain the capacity of these mutations to rescue GP(R64A).

### GP(R64A) displays increased thermostability.

Because THL and CatL display broad substrate specificities, we considered it unlikely that the change in proteolytic processing seen with GP(R64A) was due to the loss of a specific cleavage site. Instead, we postulated that R64A induces changes in GP conformational dynamics that alter its susceptibility to proteolysis. To probe these changes, we used an assay that measures the thermostability of a conformational antibody epitope in the GP base (R. H. Bortz, A. C. Wong, et al., unpublished data). Briefly, VSVs bearing GP(WT) and GP(R64A) were incubated at temperatures ranging from 46 to 80°C and then captured on an ELISA plate. GP was then detected using KZ52, a monoclonal antibody that recognizes an epitope spanning GP1 and GP2 that is lost upon being heated (19).

**FIG 7:**
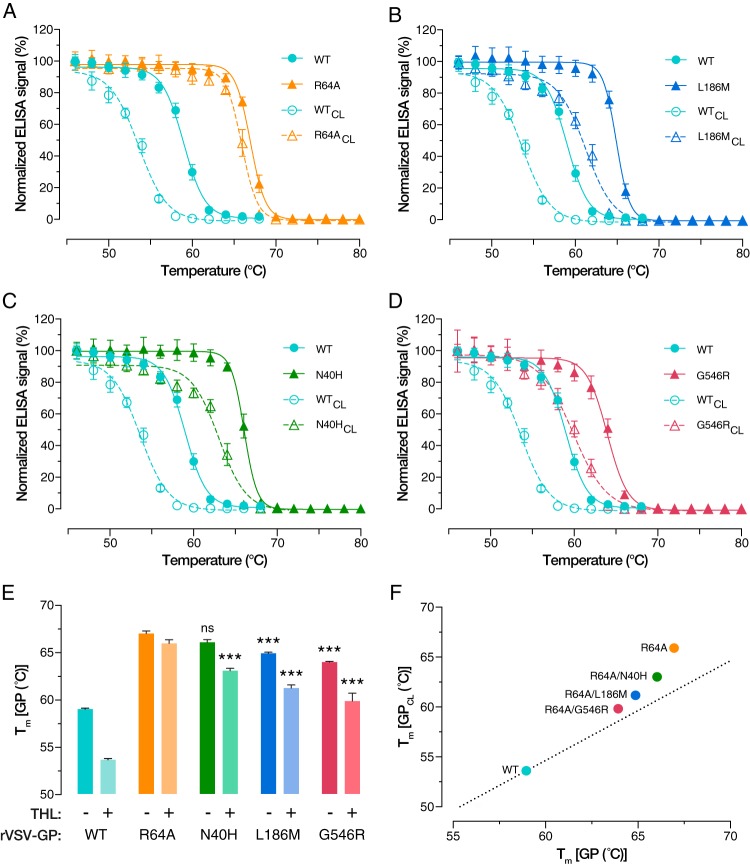
(A to D) Representative KZ52 epitope melting curves of rVSV-GP(WT) and three R64A second-site revertants. (E) *T_m_* values of THL-treated and native GPs obtained by nonlinear regression analysis. Comparing native GP(R64A) stability with that of the second-site revertants, we see a statistically significant decrease for L186M and G546R. Following THL treatment, *T_m_* values of all tested second-site revertants are significantly lower than that of the parental mutant under the same conditions. (***, *P* < 0.001, by one-way ANOVA with Dunnett’s correction for multiple comparisons). (F) Graphical representation of *T_m_* values showing the relationship between WT, R64A, and the second-site revertants. Results from nine trials from three independent experiments with three technical replicates are shown.

For GP(WT), we observed a characteristic half-maximal melt temperature (*T_m_*) of 59°C for the KZ52 epitope, which was reduced to 53°C following treatment with THL, consistent with the destabilizing effect of the GP→GP-17K cleavage. In contrast, the KZ52 epitope in GP(R64A) melted at a much higher temperature (*T_m_* = 68°C). We conclude that a single point mutation in the GP base can stabilize a GP1-GP2-bridging base epitope. Further highlighting the stability of GP(R64A) is the observation that THL treatment had only a minor destabilizing effect (Fig. 7A). These observations indicate not only that GP(R64A) is much more thermally stable than GP(WT) but also that it largely retains this phenotype following proteolysis. We likewise analyzed a subset of R64A second-site revertants, the N40H, L186M, and G546R revertants, for thermostability and found that their *T_m_* values were in the range of 64 to 66°C. Further, they were partly destabilized upon THL cleavage, with *T_m_* values reaching 60 to 63°C (Fig. 7B to E). Our findings taken together point to defects in the capacities of uncleaved and *in vitro*-cleaved GP(R64A) to initiate and complete viral membrane fusion, respectively. Although we have not yet established the precise entry-related correlates of GP’s thermostability, previous evidence that additional GP proteolysis is required at a late entry step downstream of fusion activation (6, 10, 11) raises the possibility that both upstream and downstream entry blocks encountered by GP(R64A) reflect defects in proteolysis that stem from (or relate to) its hyperstability.

### Destabilization of GP(R64A) through treatment with toremifene partially rescues infection.

**FIG 8:**
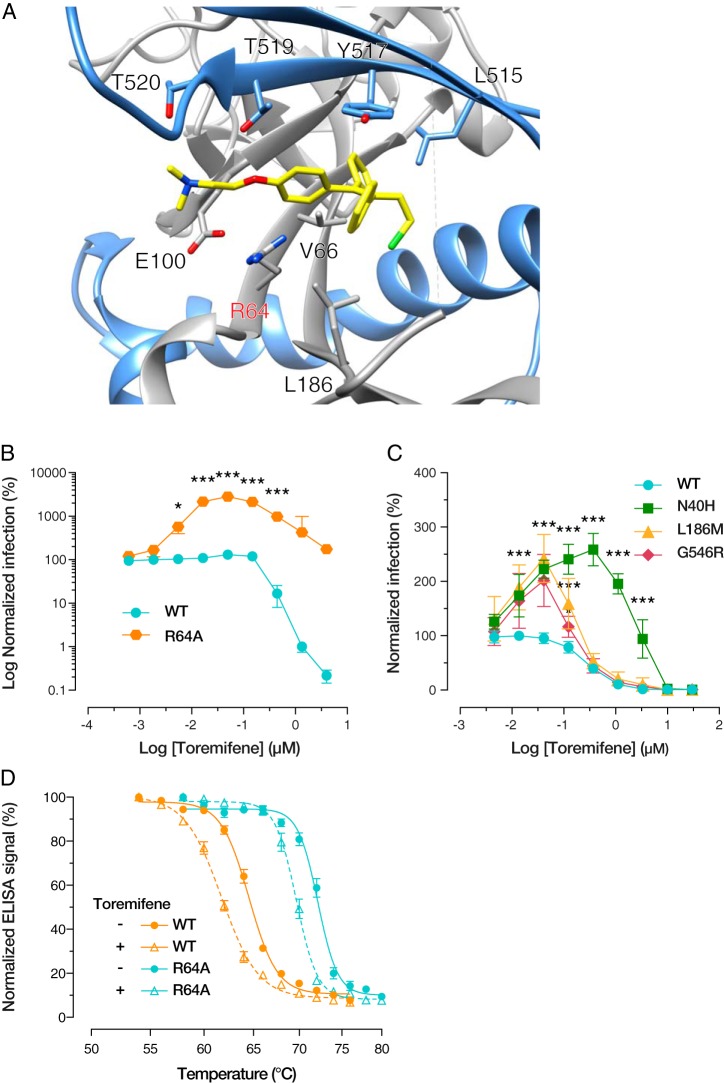
(A) Crystal structure of EBOV GP in complex with toremifene, as obtained by Zhao and colleagues (20). GP1 is gray, GP2 light blue, and toremifene yellow. (B) Relative infection levels of VSV-GP(WT) and VSV-GP(R64A) following treatment with toremifene. At subinhibitory concentrations of toremifene, VSV-GP(R64A) infection is significantly increased (*, *P* < 0.033; ***, *P* < 0.001, determined by two-way ANOVA using Šidák correction for multiple comparisons). (C) A subset of R64A second-site revertants were tested for infectivity in the presence of toremifene. All show a biphasic curve similar to that of the parental mutant but with smaller magnitude increases in infection levels. Over a range of toremifene concentrations, this increase is statistically significant compared to the infection level of GP(WT) (*, *P* < 0.033; **, *P* < 0.002; ***, *P* < 0.001, determined by two-way ANOVA using Šidák correction for multiple comparisons). Results from six trials from two independent experiments with three technical replicates are shown. (D) Representative KZ52 epitope melting curves of GP(WT) and GP(R64A) in the presence of 10 μM toremifene or a DMSO control. For both WT and mutant GP, a statistically significant decrease in *T_m_* is observed upon addition of toremifene (*, *P* < 0.033, by unpaired *t* tests).

The selective estrogen receptor modulator (SERM) toremifene has been shown to have anti-EBOV properties *in vitro* and is proposed to mediate this effect through direct binding to and destabilization of the prefusion conformation of GP (20). A crystal structure of EBOV GP in complex with toremifene reveals that the toremifene-binding pocket is located at the base of GP, between the handle of the fusion loop and the N-terminal heptad repeat region (NHR) of GP2. The toremifene molecule itself is coordinated by residues from both GP1 and GP2, including R64, V66, E100, L186, L515, Y517, T519, and T520 (Fig. 8A). Accordingly, we postulated that toremifene might enhance the infectivity of VSV-GP(R64A) by reversing the latter’s hyperstability. To evaluate this hypothesis, we incubated VSV-GP(WT), VSV-GP(R64A), and a subset of second-site revertants with a dilution series of toremifene and then exposed them to Vero cells. For VSV-GP(WT), a characteristic sigmoidal inhibition curve was seen with a half-maximal inhibitory concentration (IC_50_), closely matching previous reported values for toremifene (21). In contrast, curves for GP(R64A) (Fig. 8B) and the second-site revertants (Fig. 8C) were biphasic, with enhancement of infection occurring at sub-IC_50_ concentrations, followed by inhibition at values far greater than the IC_50_. The magnitude of enhancement correlated with the level of hyperstabilization of each mutant, with GP(R64A) maximally reaching an ∼30-fold increase in infection. The biphasic nature of enhancement followed by inhibition suggests that the stability landscape of GP has a point which allows for infection, whereas points of stability above (e.g., R64A mutation) or below (e.g., high concentrations of toremifene) are nonpermissible. We also confirmed that addition of toremifene indeed shifts the thermostability of both GP(WT) and GP(R64A) to similar degrees (Fig. 8D). Although toremifene treatment did not fully rescue the infectivity of GP(R64A) (or its thermal lability), highlighting that the effect of this mutation is likely to be multifaceted, this striking and seemingly paradoxical behavior of toremifene strongly suggests that the hyperstabilization of GP is one mechanism by which R64A arrests EBOV entry.

## DISCUSSION

A body of recent work has uncovered many features of the complex cell entry mechanism used by filoviruses, including their exploitation of (i) cell surface phosphatidylserine receptors for binding and internalization (22, 23), (ii) endocytic trafficking pathways for delivery to late endosomal compartments where viral membrane fusion can occur (2, 11, 24), (iii) endosomal cysteine proteases for programmed GP disassembly (5, 6), and (iv) the lysosomal cholesterol trafficking protein NPC1, whose recognition by a proteolytically primed form of GP in endosomes is a prerequisite for viral escape into the cytoplasm (2–4). Despite this progress, however, key questions remain. As a case in point, late entry steps leading up to viral membrane fusion remain enigmatic; the specific molecular trigger for GP fusion activation is undefined, as are the putative roles of host cysteine proteases at one or more steps downstream of GP fusion activation but prior to cytoplasmic escape (5, 6, 10).

Here, we investigated a set of GP mutants hypothesized to be entry defective by Manicassamy and coworkers (13) and identified one mutation, R64A, that abolished infection with no apparent impact on GP expression, folding, or incorporation into VSV pseudotypes. We found that GP(R64A) was highly resistant to proteolysis *in vitro*, leading us to speculate that a block at the step of CatL and CatB priming may be responsible for the lack of infection. However, the inability of THL pretreatment to rescue infection suggested the presence of a downstream block that may act alone or in addition to an upstream block to initial steps in GP proteolytic priming. Consistently with the hypothesis that R64A arrests GP-dependent entry at multiple steps, we observed distinct phenotypes for viruses bearing uncleaved and *in vitro*-cleaved GP(R64A) species in a live-cell lipid mixing assay. Specifically, whereas uncleaved VSV-GP(R64A) particles underwent little lipid mixing, cleaved mutant particles displayed a hyperfusogenic phenotype in live cells. These and other observations suggest that (i) resistance to proteolytic priming in cells renders uncleaved GP(R64A) incapable of recognizing NPC1 and/or undergoing fusion activation during entry, (ii) *in vitro* proteolytic priming relieves these upstream block(s) and affords NPC1 recognition and GP fusion activation, but (iii) despite this, cleaved GP(R64A) remains incapable of completing viral membrane fusion and mediating delivery of nucleocapsid cores into the cytoplasm, indicating the existence of one or more additional blocks at late step(s) in entry.

To obtain further insight into the complex nature of the entry blockade imposed by R64A, we isolated a set of second-site mutations that restored the infectivity of GP(R64A) to WT levels. Although these revertants grossly retained a protease-resistant phenotype *in vitro*, we nevertheless observed alterations in their dependence on cellular CatB for entry, suggesting that the second-site mutations have subtle effects on the process of GP proteolysis in cells. Interestingly, we found for the first time that mutations can not only reduce but can also enhance the CatB dependence of GP and that mutations in the β13–14 loop linking the GP base with the glycan cap can modulate CatB dependence. The latter observation, especially for mutations near the “DFF lid” motif (residues 192 to 194) at the N terminus of the β13–14 loop (14, 20), raises the possibility that the packing of this loop into the pocket occupied by toremifene and other small-molecule inhibitors (see reference 20 and below) can modulate the protease dependence of the initial GP-priming cleavages.

We next examined the behavior of viral particles bearing R64A and their second-site revertants in a novel *in vitro* GP thermostability assay (R. H. Bortz III, A. C. Wong, M. G. Grodus, H. Recht, M. C. Pulanco, R. K. Jangra, S. J. Anthony, and K. Chandran, unpublished data). We found that GP(R64A) was greatly hyperstabilized relative to GP(WT). Two additional observations correlated this phenotype with GP(R64A)’s loss of function at both upstream and downstream entry steps. First, the second-site revertant mutations that rescued GP(R64A)-mediated infection also ameliorated GP(R64A)’s hyperstability, especially upon *in vitro* cleavage. Second, we found that toremifene, a small-molecule entry inhibitor that binds into a pocket at the base of GP and destabilizes it (20), could enhance the infectivity of VSV-GP(R64A) by ∼30-fold at subinhibitory concentrations but had no such effect on VSV-GP(WT). Toremifene had intermediate effects on viral particles bearing selected GP second-site revertants.

Interestingly, despite its hyperfusogenic phenotype in cells, cleaved GP(R64A) remained hyperstable *in vitro*, suggesting that additional cleavages in cells (not recapitulated *in vitro*) reduce its stability to a degree adequate for fusion activation and/or that GP hyperstability reflects a downstream defect in the execution of GP conformational rearrangements required for the completion of viral membrane fusion and cytoplasmic escape. Although more work is needed, we speculate that R64A causes distinct blocks to GP proteolytic processing at both upstream and downstream entry steps. The former block, which can be relieved by *in vitro* cleavage, liberates the glycan cap and mucin domain from GP, affording GP-NPC1 recognition and likely reducing the threshold for GP fusion activation (as also suggested by *in vitro*-cleaved GP’s reduced thermostability, at least for the WT (Fig. 7A) (4–6, 11, 12). The latter (downstream) block is only inferred from the continued requirement for endosomal cysteine protease activity by viral particles bearing the *in vitro*-cleaved ∼17,000-molecular weight form of GP (5, 6, 10); R64A may provide a tool to further elucidate the molecular basis of this putative GP proteolytic cleavage downstream of fusion activation (11).

To our knowledge, GP(R64A) is the first hyperstabilized EBOV GP mutant to be described in the literature and may provide a valuable tool for the exploration and rational design of stability-modulating antivirals. A large body of work has previously described stability-modulating mutations in influenza A virus hemagglutinin (HA), showing that single mutations can have profound impacts on the stability of HA, as measured by changes in the pH threshold of fusion (25, 26). These mutations are often located in regions of HA that undergo large rearrangements during fusion and tend to involve ionic interactions (27). In contrast, R64 is located in a region of GP1 with no known function and no clear involvement in the large-scale rearrangements of GP2 that drive membrane fusion, suggesting a role in productive fusion activation as opposed to fusion execution. Stability-modulating mutations have also been shown to enhance the immunogenicity of HIV-1 Env immunogens (28, 29). In particular, the I559P substitution of the SOSIP immunogen has been shown to keep gp41 in a prefusion state (30). In an analogous fashion, a thermostable EBOV GP bearing R64A may have utility for the development of next-generation EBOV vaccines with improved stability. Finally, we propose that further study of the toremifene-binding pocket may yield insights into the mechanism of GP-mediated fusion. Because toremifene is thought to expel the GP DFF lid sequence at the N terminus of the β13–14 loop from its binding pocket (20), we hypothesize that R64 plays a key role in mediating the interaction between the DFF lid and the rest of GP. The R64A mutation would enlarge the cavity into which the DFF sequence inserts and thereby potentially enhance this interaction. According to this model, this may prevent cellular cathepsins from accessing the β13–14 loop (block upstream of NPC1 binding) but also hinder the timely expulsion of the DFF lid from the GP base during viral membrane fusion (block downstream of NPC1 binding).

## MATERIALS AND METHODS

### Cells and viruses.

Vero African grivet kidney cells were cultured in Dulbecco's modified Eagle medium (DMEM) (Life Technologies, Carlsbad, CA) supplemented with 2% fetal bovine serum (Atlanta Biologicals, Flowery Branch, GA), 1% penicillin-streptomycin (Life Technologies, Carlsbad, CA), and 1% GlutaMax (Life Technologies, Carlsbad, CA). Cells were kept at 37°C with 5% CO_2_ in a humidified incubator. Pseudotyped VSV bearing variant GPs was prepared as described previously (10, 15). All GPs used were based on the EBOV/H. sapiens-tc/COD/1976/Yambuku-Mayinga isolate amino acid sequence (GenBank accession number AF086833), but with a deletion of the mucin domain (residues 309 to 489) (31).

### Infection experiments.

Confluent Vero cells were infected with pseudotyped VSV bearing mutant GPs. VSVs were diluted in DMEM prior to infection. Infected cells were then maintained at 37°C for 14 to 16 h postinfection before manual counting of eGFP-positive cells or automated counting using a CellInsight CX5 high-content screening platform (Thermo Fisher Scientific, Waltham, MA) or a Cytation 5 cell imaging multimode reader (BioTek Instruments, Winooski, VT) and onboard software.

### NPC1 domain C ELISA.

NPC1 binding ELISAs were performed as described previously (4). Plates were coated with GP-specific monoclonal antibody (Mab) KZ52 (14) diluted to 2 μg/ml in phosphate-buffered saline (PBS). VSV-GP(WT) or VSV-GP(R64A) was normalized for GP content and then incubated at 37°C for 4 h in the presence of 500 μg/ml THL (Sigma-Aldrich, St. Louis, MO). The reaction was stopped by addition of 10 mM phosphoramidon. ELISA plates were blocked using PBS supplemented with 3% bovine serum albumin (PBSA; Thermo Fisher Scientific, Waltham, MA). THL-treated virus was added to blocked KZ52-coated plates and allowed to adsorb at 37°C for 1 h. After the plates were washed with 3% PBSA, a dilution series of FLAG-tagged NPC1-domain C was added and allowed to bind for 1 h at 37°C. Bound domain C was then detected using a horseradish peroxidase (HRP)-conjugated anti-FLAG antibody (Sigma-Aldrich, St. Louis, MO) and the Ultra-TMB substrate (Thermo Fisher Scientific, Waltham, MA). Half-maximal effective concentration (EC_50_) values were calculated from two independent experiments, each with three technical replicates.

### Protease inhibition and infection.

Confluent Vero cells were treated with the protease inhibitor CA-074 (Sigma-Aldrich, St. Louis, MO) or E-64 (Sigma-Aldrich, St. Louis, MO), with dimethyl sulfoxide (DMSO) as a negative control. CA-074 was diluted to 80 μM in complete DMEM, and E-64 was diluted to 300 μM in complete DMEM. Uptake of inhibitors into cells was allowed to proceed for 4 h at 37°C. In parallel, rVSV-GP(WT) as well as R64A second-site revertants were treated with THL at 500 μg/ml for 4 h at 37°C or mock treated before addition of 10 mM phosphoramidon to stop the reaction. Inhibitor-treated cells were then infected with THL- or mock-treated rVSV-GPs for 1 h at 37°C before addition of 20 mM NH_4_Cl to prevent additional rounds of infection. Following incubation at 37°C for ∼14 h, cells were fixed using paraformaldehyde, stained using Hoechst 33342 nuclear stain, and then scored for infection. Mean results of four independent experiments, each comprising two technical replicates, are shown.

### *In vitro* protease conditions.

VSV-GP(WT) or -GP(R64A) was incubated with THL at 250 μg/ml for 30, 60, or 120 min at 37°C before the reaction was stopped by addition of 10 mM phosphoramidon. Cleavage products were then deglycosylated through treatment with 250 U PNGase F (New England Biolabs, Ipswich, MA) for 16 h at 37°C. In order to achieve complete cleavage, concentrations of 500 μg/ml THL and incubation times of 4 h at 37°C were used instead.

### SDS-PAGE and Western blotting.

Deglycosylated samples containing GP cleavage products were analyzed by SDS-PAGE followed by Western blotting for GP1 using rabbit polyclonal sera recognizing a peptide (TKRWGFRSGVPPKVV) overlapping the receptor-binding site (5). IRDye 680LT goat anti-rabbit IgG secondary Ab (LI-COR, Lincoln, NE) was used at a dilution of 1:10,000, and the final blot was then imaged using a LI-COR Fc fluorescent imager.

### Virus labeling.

To detect viral membrane fusion within cells, pseudotyped VSV particles were labeled as previously described (32). Briefly, concentrated virus (1 mg/ml) was incubated with 50 μM DiD while being agitated for 1 h at 4°C. Unincorporated dye was removed by ultracentrifugation of the virus over a sucrose cushion. Labeled virus was aliquoted for single use and stored at −80°C. THL-treated versions of VSVs with completely cleaved GPs were prepared as described above.

### Live-cell microscopy.

Live imaging was performed with a Zeiss AxioObserver.Z1 widefield epifluorescence microscope equipped with a heated environmental enclosure maintained at 37°C, a 40×/1.35-numerical-aperture oil immersion objective, and a DAPI (4′6-diamidino-2-phenylindole)-green fluorescent protein (GFP)-Texas Red-Cy5 filter set as previously described (11). U2OS cells were seeded onto fibronectin-coated 35-mm coverslip dishes (MatTek, Ashland, MA) to be at approximately 70% confluence for imaging the next day. For expression of cellular markers, cells were transduced with the CellLight vector (Life Technologies, Carlsbad, CA) for early (GFP-Rab5) and late (GFP-Rab7) endosomes (GFP-Rab5) 18 h prior to being imaged according to the manufacturer’s instructions. Dishes were chilled several minutes on ice before DiD-labeled virus was spinoculated onto monolayers at 6°C and 1,500 × *g* for 20 min. Unbound virus was removed by five washes with cold PBS, and 500 μl cold imaging buffer (140 mM NaCl, 2.5 mM KCl, 1.8 mM CaCl_2_, 1 mM MgCl_2_, 20 mM HEPES, 5 mM glucose, 2 mg/ml Hoechst 33342, and 2% fetal bovine serum, pH 7.4) was added to cover the cells. The dish was immediately mounted on the microscope objective and focused, and addition of 1.5 ml warm imaging buffer to the dish marked the start of experiments (time zero). Images were acquired every 10 s for 2 h using a single Z-section, which encompassed the large majority of cell-associated virions.

### Data analysis.

Image analysis and single-particle tracking were performed using Volocity 6.3 software (PerkinElmer) as previously described (11). Puncta were thresholded by size and initial fluorescence intensity in order to exclude viral aggregates. Virions were considered colocalized with cellular markers if the puncta cotrafficked with at least 80% overlap of signals and if the intensity of the marker exceeded the background fluorescence by at least 30%. Mean measurements plus standard deviations (SD) were derived from three independent experiments, unless otherwise indicated. Statistical significance was established by Student's *t* test or one-way analysis of variance (ANOVA) with a *post hoc* Tukey test (**, *P* < 0.01; ***, *P* < 0.001; ****, *P* < 0.001).

### GP thermostability assay.

rVSV-GP(WT), GP(R64A), and R64A second-site revertants were treated with THL at 500 μg/ml for ∼14 h at room temperature or mock treated before addition of 10 mM phosphoramidon to stop the reaction. Cleaved and uncleaved virus was diluted in PBS and then heated at a range of temperatures spanning from 42 to 80°C (the actual range for each virus is indicated in the text) for 10 min, followed by a drop to 4°C using a thermal cycler (Applied Biosystems, Foster City, CA). After cooling, heated virus was directly captured onto highly binding 96-well half-area ELISA plates (Corning, Corning, NY.) Plates were then blocked using 3% BSA in PBS. GP was detected with KZ52, a conformation-specific anti-EBOV GP human monoclonal antibody. Antibody bound to GP was detected with an anti-human antibody conjugated to HRP (EMD Millipore, Burlington, MA) and the Ultra-TMB substrate (Thermo Fisher, Grand Island, NY). All binding steps were carried out at 37°C for 1 h. Binding curves were generated using Prism (nonlinear regression, variable slope [four parameters]; GraphPad Software, La Jolla, CA). *T_m_* values were calculated from three independent experiments, each with three technical replicates. GP thermostability in the presence of toremifene was assessed as described above, with the addition of 10 μM toremifene (Sigma-Aldrich, St. Louis, MO) or 0.1% DMSO as a control during the heating step. All thermostability experiments in the presence of toremifene were carried out in PBS adjusted to pH 5.5 in order to achieve full toremifene activity.

### Rescue of rVSV-GP(R64A) second-site revertants.

rVSV-GP(R64A) was rescued from cDNA as described previously (10, 18). Briefly, 293FT cells were transfected with plasmids encoding proteins of the VSV replication machinery (N, P, and L) and the VSV glycoprotein (G), as well as a VSV genomic plasmid with EBOV GP(R64A) in place of the orthologous glycoprotein. The A64 codon (GCC) was chosen to disfavor reversion to WT. The genomic plasmid also includes eGFP in the first frame as an infection marker. After passaging of virus-containing supernatants on Vero cells, viral clones were isolated by plaque purification. A total of 40 plaques from five independent rescues were isolated and sequenced. All isolated viruses retained the A64 mutation and also gained one or two second-site mutations (Table 1).

### Toremifene inhibition/enhancement of infection.

rVSV-GP(WT), VSV-GP(R64A), and rVSV-GP(R64A) second-site revertants were incubated with a dilution series of toremifene (Sigma-Aldrich, St. Louis, MO), starting at 30 μM (second-site revertants) or 4 μM [VSV-GP(R64A)], at room temperature for 2 h. The virus-drug mixture was then added to Vero cells and cultured as described above, and infection was allowed to proceed at 37°C for 1 h before addition of 20 mM NH_4_Cl. Following 14 h of incubation at 37°C, infection was scored by automated counting of GFP-positive cells as described above. After normalization of infection levels using DMSO-treated virus as a control, significance was calculated from two independent experiments, each comprising three technical replicates, using two-way ANOVA with the Šidák correction for multiple comparisons (*, *P* < 0.033; **, *P* < 0.002; ***, *P* < 0.001).
